# Editorial: Insights in pediatric endocrinology: 2022

**DOI:** 10.3389/fendo.2023.1233451

**Published:** 2023-06-14

**Authors:** Madhusmita Misra, Sally Radovick

**Affiliations:** ^1^ Division of Pediatric Endocrinology, Massachusetts General Hospital, Boston, MA, United States; ^2^ Department of Pediatrics, Robert Wood Johnson Medical School, Rutgers, The State University of New Jersey, New Brunswick, NJ, United States

**Keywords:** pediatric endocrinology, puberty, growth, hypothalamic amenorrhea, congenital adrenal hyperplasia

This special edition Research Topic was designed to highlight the progress made in Pediatric Endocrinology in the past year and to provide a thorough overview of the field. This Research Topic includes novel developments and discoveries, discusses current challenges, and provides new insights and perspectives that will guide the field into the future.

A research manuscript by Yang et al. uses chest CT to determine body composition and the relationship between the growth hormone (GH)/insulin like growth factor-1 (IGF-1) axis and muscle density in children with short stature. In a large retrospective study that included 297 children with a mean age of 10 years admitted during the COVID-19 pandemic, records were assessed for serum GH, IGF-1 levels, and two GH stimulation tests. The authors determined that peak GH is correlated with the fat mass index, and IGF-1 SDS with the skeletal muscle index. These data indicate that the GH/IGF-1 axis uses different mechanisms to regulate muscle and fat development and metabolism. The authors point to several limitations of the study. These include the cross-sectional design, inability to predict the risk of metabolic diseases, confounding variables that were not analyzed, including family income, exercise intensity, and dietary habits, and an inability to show a gender difference because the majority of children were prepubertal. Lastly, as the study was conducted in China, the findings may not be readily generalizable to other populations or ethnicities.

Although extensively used as an effective screening tool for growth hormone deficiency (GHD), bone age (BA) readings are time-consuming and can be highly variable between clinicians. Several AI systems have already been developed to assess BA yielding higher accuracy and improved time efficiency compared to manual assessment. Zhang et al. hypothesize that BA assessment gaps exist between junior and senior-level clinicians and explore the subject of using AI to assess BA in China among children with GHD. The study seeks to show that AI-assisted BA interpretation improves precision and decreases variability for junior pediatric endocrinologists during the treatment course in children with GHD. Since the classic methods used to evaluate BA were developed based on a Caucasian population (Greulich-Pyle atlas and Tanner-Whitehouse), an alternative method developed by the Chinese Bone Development Survey Group is used in the Asian population, China 05 (CH05). In the study, 290 BA radiographs from 52 children were read by senior pediatric endocrinologists, and their consistent results were regarded as the gold standard. Two junior pediatric endocrinologists assessed the BA with and without assistance from the AI-based BA evaluation system. 20% of the images assessed by the junior pediatric endocrinologists were randomly selected and similarly re-evaluated. The performance of the junior pediatric endocrinologists improved, with the precision increasing from about 10% to over 91% using AI assistance. During GHD treatment, the longitudinal difference significantly decreased and inter-rater effect was no longer present when using AI-based BA evaluation. One of the limitations was the lack of involvement of senior pediatric endocrinologists to further validate the usage and clinical value of AI-based interpretations. Clearly, the use of AI technology will improve the precision and efficiency of BA assessments.

Mason and Rogol give a historical perspective on growth and pubertal development in children with cystic fibrosis (CF). They trace advances in pulmonary and nutritional management to improvements in the growth and development of children with CF. The authors cite multiple etiologies of impaired growth in children with CF, including malabsorption, reduced caloric intake, increased resting energy expenditure, glucocorticoid exposure, systemic inflammation, and a role for the CFTR genotype. They describe studies showing the evolution of increasing weight and height z-scores, with greater increases in weight than height. This results in some children with CF meeting criteria for overweight or obesity. The etiology is unclear and may be due to a direct effect of CFTR on the GH-IGF-1 axis. Although the literature clearly shows an association between nutritional status and pulmonary function, some recent data demonstrate an association between height in early childhood and long-term pulmonary function independent of nutritional status. They conclude with the importance of identifying factors that impact growth impairment in early life and follow growth in children receiving CFTR modulator therapy. The critical role of nutritional guidelines to optimize pulmonary function and linear growth is emphasized while also preventing obesity and its comorbidities. This is crucial at a time when early detection and modulator therapies present great promise.

Pedreira et al. focus on the role of a hypoestrogenic state of functional hypothalamic amenorrhea (FHA) on bone health. The manuscript reviews the pathogenesis of FHA induced bone changes, including the low estrogen conditions, exercise and anorexia nervosa, that result in compromised skeletal health. It also describes treatment strategies to mitigate bone loss. The sections related to the determinants of bone health in FHA are comprehensive and include contributions from the reproductive, growth, and adrenal axes and appetite-regulating hormones. The treatment strategies include a review of the controversies in the field related to whether a critical weight is necessary for the resumption of menstrual cycles. In a related section, strategies for managing bone density are discussed and include a table with a comprehensive listing of therapeutic interventions. A small part of the article is devoted to neuropsychiatric outcomes of low estrogen states, including cognitive function, emotion and mood, and eating behaviors. It finishes with a section relating to hormonal correlates.

Baskaran et al. report higher scores of anhedonic depression and anxiety in hypoestrogenic amenorrheic athletes compared with normoestrogenic eumenorrheic athletes 14-25 years old. They also demonstrate higher caudate volumes in amenorrheic vs. eumenorrheic athletes, with lower activation during reward anticipation in the right caudate in amenorrheic athletes. The latter is suggestive of a blunting of reward processing in the striatum in conditions of estrogen deficiency indicating that estrogen status may impact how we process reward.

In their review of the impact of glucose metabolism on the developing brain, Cacciatore et al. discuss studies that demonstrate deleterious effects of both hypoglycemia and hyperglycemia on brain development, cognitive function (including an impact on intelligent quotient, learning, memory and executive function). This is because glucose is an essential substrate for brain development and functioning. They also review functional MRI data, including findings of alterations in brain structure and function even after a single episode of diabetic ketoacidosis.

There is a dearth of information on biomarkers for complications of type 1 diabetes mellitus in children. Gong et al. describe a novel biomarker, alpha-klotho, for diabetic nephropathy, a major cause of end-stage renal disease, in children with type 1 diabetes mellitus. Although alpha-klotho (KL), a co-receptor for fibroblast growth factor (FGF) 23, which is regulated by the miRNA miR-192, is lower in mouse models and adults with chronic kidney disease, data are limited in children. The investigators studied 79 pediatric patients with type 1 diabetes for 7.2 ± 3.9 years with a 2-year average HbA1c of 8.0 ± 1.3. They found that KL was inversely correlated with diabetes duration and HbA1c, indicating its potential role in glycemic control. Serum miR-192 was negatively associated with KL among children with a prolonged duration of diabetes (≥12 years). A mechanistic approach to understanding the role of miR-192 and KL expression in a cell culture model demonstrated overexpression of miR-192, downregulated cellular KL mRNA and protein levels as well as decreased KL levels in the media. Using a reporter assay, a miR-192 mimic reduced the activity of a reporter. Additional studies showed an increase in oxidative stress and expression of inflammatory and senescence markers. These data suggest that KL is a direct target of miR-192 and implicate oxidative stress as the mechanism of renal disease. They conclude that miR-192 and/or KL levels could serve as early biomarkers for diabetic nephropathy in children with type 1 diabetes.

This Research Topic includes three papers on precocious puberty and one on congenital adrenal hyperplasia. Prosperi and Chiarelli review data from multiple studies from Italy, China, Turkey and India that report an increase in cases of central precocious puberty and rapidly progressive puberty during the COVID-19 pandemic, sometimes, but not always related to an increase in BMI from the more sedentary lifestyle during the lockdown. Most papers do not provide documentation of SARS-COV-2 infection in children presenting with early or rapidly progressive puberty, and the authors speculate whether there may be a direct effect of the virus, or an indirect effect (through psychological factors) on pathways regulating GnRH secretion.

The review by Calcaterra et al. addresses the ‘hot topic’ of the gut microbiome and sex steroids, and they explore interactions, potentially bidirectional, between the gut microbiome and sex hormones, differences in boys vs. girls during pubertal development and during female precocious puberty. Presently, the evidence on the interaction between gut microbiota and sex hormones remains limited in pediatric patients. The authors begin by reviewing the basic principles of puberty and precocious puberty, followed by a summary of the role of the microbiome in known health and disease states. They focus on known associations between the gut microbiome and obesity and relate this to the earlier puberty seen in obese children. They then explore known interactions between the gut microbiome and sex steroids, referred to as the sex-hormone-gut microbiome. Several manuscripts are referenced, which provide evidence that the diversity of the gut microbiome changes through pubertal development and alterations may occur in girls with central precocious puberty (CPP). The authors call for additional research to increase our understanding of the relationship between sex hormones and the gut microbiome. They summarize by stating that further clarification of the interactions between sex hormones and the gut microbiome may lead to microbiota-targeted therapies in pubertal disorders.

Yoo et al. report on the efficacy and safety of the 22.5 mg 6-monthly triptorelin pamoate formulation in suppressing puberty and improving adult height prediction in 33 girls and 9 boys with central precocious puberty. Six-monthly depot gonadotropin releasing hormone analogs allow clinic visits to be spaced out for patients with central precocious puberty and reduce patient burden. Itonaga and Hasegawa review current knowledge regarding monitoring of treatment of pediatric patients with 21-hydroxylase deficiency, and discuss the pros and cons of biochemical serum (17-hydroxyprogesterone, androstenedione and ACTH) and urine (pregnanetriol and other steroid metabolites) testing. At this time serum testing is preferred because it is less expensive, more standardized, and well-established in clinical care.

Finally, Dacal et al. present interesting data from prepubertal and pubertal boys with hematological malignancies before initiation of chemotherapy and show lower levels of inhibin B and anti-Mullerian hormone with low or inappropriately normal FSH levels compared to a reference population, with lesser involvement of the LH-Leydig cell compartment (12). Following three months of chemotherapy, the authors report high levels of FSH and LH levels with persistently low levels of inhibin B and anti-Mullerian hormone, but normalization of testosterone. The authors posit occurrence of a primary testicular dysfunction with an associated functional central hypogonadism before treatment initiation (when general markers of health status are also low), with improvement in hypothalamic-pituitary function (but persistence of testicular dysfunction) following three months of chemotherapy, when markers of general health are improved.

The Research Topic thus covers a range of novel topics and comprehensive reviews addressing important issues in the field of pediatric endocrinology today. As always, more research is necessary to answer hitherto unanswered questions, and we hope that perusal of this Research Topic stimulates ideas for future research, including collaborative research.

## Author contributions

All authors listed have made a substantial, direct, and intellectual contribution to the work and approved it for publication.

